# Assessing the Environmental Impacts, Condition and Sustainability of Mountain Biking Trails in an Urban National Park

**DOI:** 10.1007/s00267-024-02029-6

**Published:** 2024-08-17

**Authors:** Isabella Smith, Catherine Marina Pickering

**Affiliations:** 1 Centre for Planetary Health and Food Security, Griffith University, Gold Coast, QLD Australia; 2 School of Environment and Science, Griffith University, Gold Coast, QLD Australia

**Keywords:** Trail impacts, User-created trails, Geographic information systems, Rapid trail assessment, Recreation ecology

## Abstract

Mountain biking is a popular recreational activity in natural areas, with thousands of formal trails designed, constructed and maintained by land managers. Increasingly, there are also rising numbers of informal trails created by riders. A challenge for land managers is identifying, assessing, and then mitigating environmental impacts created by trails, including in protected areas. Here we assessed mountain biking trails in a large, popular national park on the Gold Coast, Australia, addressing the currently limited research comparing the extent, environmental impacts, condition and sustainability of these trails. Impacts from the 31.4 km of formal and 33.7 km of informal trails through the forests in Nerang National Park (1659 ha) included soil erosion (16.48 m^3^) and loss of vegetation along and adjacent to the trails (90,955 m^2^). Formal trails were six times more popular and wider on average (1.1 m vs 0.7 m) than informal trails, but less incised than informal trails (4.6 cm deep vs 6.3 cm). Generalised Linear Models showed that Trail Grade, slope and alignment best-predicted trail condition, highlighting the importance of good trail design in minimising trail impacts. It is recommended most of the informal trails are closed and rehabilitated, as they were not well-designed, increase fragmentation and have environmental impacts, with some traversing ecologically sensitive areas. In addition, some formal trails need to be upgraded to deal with erosion and other impacts. More broadly, the increasing demand for mountain biking must be addressed, including exploring opportunities to promote areas outside of national parks while minimising environmental impacts and other challenges associated with the creation and use of informal mountain bike trails in protected areas.

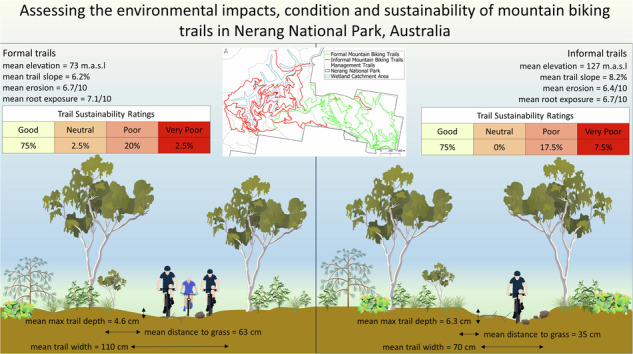

## Introduction

Protected areas are globally important for the conservation of nature and the provision of a wide range of ecosystem services (Worboys et al. 2015; Marion 2023). Within protected areas, nature-based recreation and tourism are the most commonly permitted activities with a wide range of benefits for visitors (Leung et al. 2011; Monz et al. 2020). With increasing visitation, particularly to urban protected areas, it is important to monitor who is using them, for what activities and why (Hammitt et al. 2015; Jurado Rota et al. 2019). It is also important to assess the sustainability of any infrastructure in the parks used for these activities and their environmental impacts (Norman et al. 2019; Farías-Torbidoni et al. 2023). Recreational trails are among the most common type of infrastructure in protected areas and facilitate popular activities such as hiking, trail running and mountain biking (Ballantyne et al. 2014; Farías-Torbidoni et al. 2023; Marion 2023; Smith et al. 2023). This includes formal trails designed, constructed, maintained and promoted by land managers (Webber et al. 2004; Carsten 2023; Marion 2023). These trails should provide positive and safe experiences and health and wellbeing outcomes for visitors while minimising negative environmental and cultural impacts (Webber et al. 2004; Marion 2023). The use of trails in protected areas can also contribute to local economies and funding for conservation (Pelling and Jones 2021; Marion 2023).

The design, construction and management of trails need to take into consideration a range of factors, such as the intended activity type and amount of use, as well as seasonal, climate and environmental factors (Tomczyk 2011; Marion and Wimpey 2017; Marion 2023). They should also be designed to minimise fragmentation and concentrate disturbance in areas with more resilient topography, soils, vegetation and wildlife (Wang and Ng 2024). Factors such as the width, surface type and alignment of trails also need to be considered in relation to the site and desired use (Webber et al. 2004; Tomczyk 2011; Marion and Wimpey 2017). For instance, trails running perpendicular rather than parallel to slopes can minimise water running directly along trails, thereby reducing erosion and increasing safety for visitors (Webber et al. 2004; Tomczyk et al. 2017; Carsten 2023; Spernbauer et al. 2023).

All trails have environmental impacts, even those that are designed using standards developed by government agencies and industry groups (Ballantyne and Pickering 2015b). Direct environmental impacts caused by trails include changes to: (1) soils, such as compaction and erosion (Tomczyk 2011; Barros et al. 2013; Marion and Wimpey 2017), (2) vegetation, including removal, trampling and changing structure, composition and function (Bayfield 1979; Pickering and Hill 2007; Ballantyne and Pickering 2015b; Rusterholz et al. 2021), (3) water, including changing water flows, damaging riparian vegetation and increased siltation (Kidd et al. 2014; Hammitt et al. 2015), (4) animals, such as altering behaviour and reducing habitat quality (Naylor et al. 2009; Larson et al. 2016) and (5) ecosystems through fragmentation of species habitat (Ballantyne et al. 2014; Lucas 2020; Monteiro and Cabral 2022). There are also indirect environmental impacts of trails, including altering soil chemistry (Malmivaara-Lamsa et al. 2008), changing microclimates on and near trails (Malmivaara-Lamsa et al. 2008; Marion 2023) and introducing weeds, pathogens and rubbish (Ansong and Pickering 2014; Pickering 2022). These environmental impacts are exacerbated when trails are not designed or maintained correctly, as is the case with many informal trails (Leung et al. 2011; Havlick et al. 2016; Marion 2023).

Informal trails are created by visitors to natural areas and occur for many reasons, including to extend existing trails, provide new opportunities, avoid hazards and reduce crowding and conflict with other trail users (Newsome and Davies 2009; Leung et al. 2011; Wimpey and Marion 2011; Ballantyne and Pickering 2015b; Havlick et al. 2016). There is less research on informal trails compared to formal trails, in part because they can be more difficult to find and their use can vary dramatically (Spernbauer et al. 2023). A 2015 literature review found that although there has been less research on informal trails compared to formal trails, the environmental impacts of informal trails were often found to be more severe than for well-designed formal trails (Wimpey and Marion 2011; Ballantyne and Pickering 2015a). This is of particular concern where there are large networks of informal trails created by visitors in many protected areas (Havlick et al. 2016; Spernbauer et al. 2023).

Mountain biking is a popular outdoor recreational activity with increasing participation in many regions, including North America (Monz and Kulmatiski 2016; Martin and Butler 2021), Europe (Campelo and Nogueira Mendes 2016; Arvidsen et al. 2023) and Australia (Hardiman and Burgin 2013; Australian Government 2023). In Australia, the number of people mountain biking has more than doubled from ~200,000 in 2016 to almost 500,000 in 2022 (Australian Government 2023). Research into the environmental impacts of mountain biking is also increasing, but still lags behind that for other recreational activities such as hiking (Pickering 2022). Many studies have found that environmental impacts of mountain biking per pass or per metre of trail can be similar to or slightly greater than those from hiking (Havlick et al. 2016; Evju et al. 2021; Jula and Voiculescu 2022; Pickering 2022). However, as mountain bikers often travel further, faster and go for longer than people undertaking other trail-based activities, their environmental impacts can affect larger areas (Norman et al. 2019). Furthermore, the rapid spread of e-mountain biking is increasing who goes riding, when and for how far, potentially exacerbating existing environmental impacts from traditional pedal-powered mountain biking (Kuwaczka et al. 2023; Taylor et al. 2023).

In response to a range of factors, including the desire for additional places to ride, extensive networks of informal mountain biking trails have been constructed by riders, particularly in urban protected areas (Ballantyne et al. 2014; Ballantyne and Pickering 2015b; Spernbauer et al. 2023). Technical Trail Features (TTF) are also popular with mountain bikers to increase the thrill and challenge of trails with unauthorised TTF being built by users along with those officially provided by land managers (Newsome and Davies 2009). These informal trails and features can contribute to safety risks and increase environmental impacts when they are not well designed, constructed and maintained (Newsome and Davies 2009; Ballantyne and Pickering 2015a). For those managing natural areas, including national parks popular with mountain bikers, it is critical to know the nature, extent, condition and environmental impacts of formal and informal trails (Ballantyne and Pickering 2015b; Evju et al. 2021).

The aim of this study was to assess the environmental impacts and condition of mountain biking trails in a popular urban national park. This involved comparing formal and informal mountain biking trails to see if they differ in impacts as well as assessing what factors were associated with environmental impacts, poor trail condition and less sustainable trails. It was hypothesised that: (1) both types of trails would have impacts, but (2) informal trails would have more severe impacts, and (3) that slope, alignment and higher use would be associated with greater impacts and worse trail conditions. The study also provides methodology that can be applied by land managers to assess trail conditions, including demonstrating how desktop and field assessments can be combined, and what factors may be the most important to assess.

## Methods

### Study Site

**Fig. 1:**
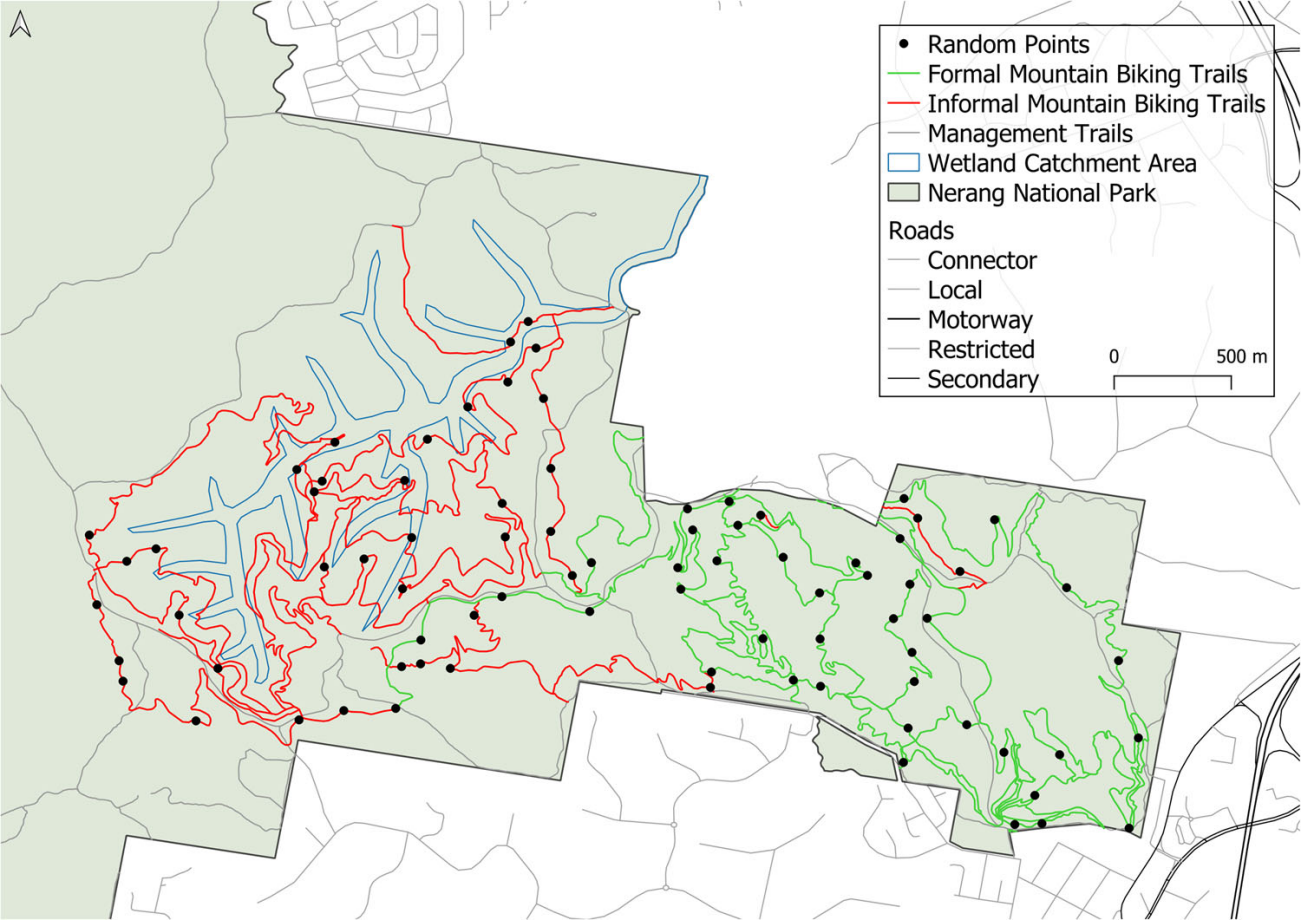
Location of formal and informal mountain biking trails, randomly allocated sampling points and wetland catchment area (Queensland Government 2005) in Nerang National Park, Australia. Management trails are also shown, although they were not sampled

Nerang (Ngarang-Wal) National Park is a large (1659 ha), IUCN category II protected area on Kombumerri country within the city of the Gold Coast (population 634,000) (City of Gold Coast 2021), located in the subtropics of South-East Queensland, Australia. This area was previously selectively logged as a State Forest, but between 2007 and 2009 it was converted to a National Park, reflecting its biodiversity values (Pickering and Rossi 2016, Lovegrove-Walsh 2021). The large urban park is dominated by *Eucalyptus* forest (Smith et al. 2023) and contributes to the conservation of 23 threatened species, including the koala, glossy black cockatoo and greater glider (Queensland Government 2022) along with three vegetation communities, known as Regional Ecosystems, that are listed as ‘of conservation concern’ (Queensland Government 2022). There are no permanent watercourses in Nerang National Park, however, the centre of the Park is part of the catchment for the Coombabah Wetlands which is of high conservation value and listed with RAMSAR (Queensland Government 2005) (Fig. 1). Much of the Park is hilly and steep (elevation range from 18 m to 235 m above sea level), particularly in the west and central parts of the Park, whilst the south-east corner, where the main entrance is, is relatively flat.

Nerang National Park supports a range of recreation activities including hiking, mountain biking and horse riding. Mountain biking is the most popular activity (Smith et al. 2023), with a mixture of single and mixed-use formal trails promoted for mountain biking in the Park (Fig. 1) (Queensland Government 2022). This includes technically challenging single-direction mountain biking trails (Black Diamond), some of which were constructed for international competitions, along with intermediate (Blue) and beginner (Green) mountain biking trails combining for a total of 32 km of formal trails across the Park. These trails are mainly bare earth with some rock and a few short sections of boardwalk constructed over steeper gullies. In a few places along both formal and informal trails, TTFs such as rock gardens and different types of jumps have been created, all using natural materials. Five of the formal trails were machine built and hand finished, with a few TTF sections in other trails also machine built (Rosewell, personal communication 2024). The rest of the formal trail network was originally informal trails hand built by users and integrated into the formal trail network after the area became a National Park between 2007 and 2009 (Rosewell, personal communication 2024). There are some wider mixed-use trails in the Park that are mostly used for access and fire control, but mountain biking, hiking and horse riding are also permitted on these management trails. In addition to the trails designed, constructed and maintained by the Park agency, there is an extensive network of informal mountain biking trails extending beyond the formal trail network (Fig. 1) (Rosewell, personal communication 2024). These informal trails are all hand built by visitors to the Park (Rosewell, personal communication 2024). Information about the formal trail network is available to visitors on government websites (Queensland Government 2022), and signage within the Park, while information about both formal and informal trails is also available to visitors from online fitness platforms such as Trailforks and Wikiloc (Smith et al. 2023; Trailforks 2023).

### Mapping Mountain Biking Trails

Prior to field work, the formal and informal mountain biking trails in the Park were mapped in QGIS (version 3.18) using data from the Queensland Parks and Wildlife Services, the government agency responsible for managing the Park, as well as Volunteered Geographic Information from the fitness platform Trailforks. Initially, 27 formal trails and 44 informal trails were identified. However, when the Queensland Parks and Wildlife Services conducted a ‘health check’ on trails in the Park in early 2023, they identified two additional informal mountain biking trails and found that some other informal trails were no longer being used (Rosewell, personal communication 2023). The land managers also closed several informal trails over the course of 2022 and 2023 (Rosewell, personal communication 2023). Therefore, in late 2023 there were 26 formal and 28 informal active mountain biking trails in the Park covering a total of 65 km (Fig. 1).

### Assessing Mountain Biking Trails

Mountain biking trails can be assessed using a combination of desktop data and field data (Ballantyne et al. 2014; Ballantyne and Pickering 2015b). Desktop data can include the topography of the site, soil and vegetation types, conservation value, as well as types of trails and their use. Field work can collect quantitative data at points along trails including data about soil erosion and compaction and impacts on vegetation on and adjacent to trails (Ballantyne and Pickering 2015b). The condition of trails either side of the point can also be assessed using trail condition class assessment protocols (Ballantyne and Pickering 2015b).

Environmental impacts and condition of trails in Nerang National Park were assessed at 40 points on formal and 40 points on informal mountain biking trails. Points were selected using the Random Points on lines tool in QGIS (Fig. 1). Prior to field work, online data relating to seven variables were obtained for each point (Table 1). The elevation and slope for each point were obtained using a Digital Elevation Model (Queensland Government 2000) and the QGIS function Sample raster layer. The type of vegetation (Regional Ecosystems) and soils at each random point were also obtained from the Queensland Government in the form of publicly available .gpx spatial files (Queensland Government 2023). Data about trail popularity, Trail Grade and difficulty level were obtained from Trailforks (2023).

**Table 1:** Details of desktop and field data collected for formal and informal trails in Nerang National Park, including the method/source of data for each trail point

	Variable	Method/source of data
Desktop data		
1	Elevation	Digital elevation model in QGIS
2	Landform slope	Digital elevation model in QGIS
3	Regional Ecosystem	Queensland Government layer in QGIS
4	Soil type	Queensland Government layer in QGIS
5	Difficulty rating	From Trailforks
6	Popularity of trail	From Trailforks from April to September 2023
7	Trail Grade	Average slope of the trail across the whole trail
Field data		
8	Trail Slope Alignment	Using a compass, the smallest difference in bearings between the prevailing landform slope (landform grade/aspect) and the trail’s alignment
9	Trail slope	Measure slope of trail surface
10	Width	Measure width of trail
11	Max. depth and point depth taken	Measure maximum depth of trail and where it was along width
12	Distance to strata layer	Minimum distance in straight line from the edge of the trail to nearest ground cover, shrub, tree
13	Soil compaction	Penetrometer measuring compaction
14	Canopy cover	Images were taken using the camera on an iPhone and then analysed using ImageJ
15–20	Trail condition assessment	Scale of 1–10 for erosion, root exposure, vegetation die-off, altered hydrology and presence of invasive species and overall trail condition 50 m in either direction along the trail from each point
21	Technical Trail Features	Record presence/absence as well as type and number of TTF 50 m either side of each point

*QGIS* Quantum Geographic Information System

In the field, additional data were collected for each point in September and October 2023. Environmental data included trail width, depth, trail slope, alignment (Trail Slope Alignment = TSA), distance to nearest ground cover, shrub and tree and canopy cover images (Table 1). Data on the condition of the trail were collected at the same time including visual assessments of erosion, root exposure, vegetation die-off, altered hydrology and presence of invasive weed species (Table 1). This involved ranking the trail for 50 m in both directions along the trail from each point by comparing the condition of the trail and trail verges (3 m either side of trails) to adjacent areas >3 m from the trail. For each variable a scale of one to ten was used where one represented severe degradation and/or impact, and ten represented little to no impact. The scale for each category captured both the frequency and extent of impacts, meaning that observed impacts of high severity but low frequency could still be scored relatively high. These were then averaged to get an overall condition of the trail near each point (Table 1). In addition, the presence or absence of TTFs as well as the number and type of TTFs 50 m along the trail in either direction from each point were also recorded (Table 1). To analyse changes in canopy cover between on-trails and off-trails, the photos taken in the field were processed in the open-source, java-based programme ImageJ (Abràmoff et al. 2004). Photos were binarised in the programme to produce an image where the vegetation becomes black, and background becomes white. The Measure %Area function was then used to determine the percentage of the image in black.

The desktop and field data were also used to generate sustainability scores. Specifically, trails were categorised based on their Trail Grade and TSA as Good, Neutral, Poor or Very Poor using the Trail Sustainability Ratings (TSR) criteria developed by Marion and Wimpey (2017). Trail Grade can either be calculated as an average overall for trails or for trail segments (Webber et al. 2004). Trails in Nerang National Park alternatively ascend and descend, so Trail Grade was calculated for segments then averaged out over the full trail for each trail surveyed (Webber et al. 2004; Marion and Wimpey 2017). The International Mountain Biking Association considers that an average Trail Grade of 10% or less is sustainable in relation to soil erosion and visitor safety (Webber et al. 2004; Marion and Wimpey 2017). However, this 10% guideline is general, with factors such as soil type, climate, length of trail, use and primary activity influencing what Trail Grade may be sustainable in specific settings (Marion and Wimpey 2017). The TSA is measured at a single point using a compass and is the smallest difference in angle between the landform slope (aspect) and the trail alignment (Marion and Wimpey 2017). Higher trail slope alignment values (between 46^o^ and 90^o^) are more sustainable as water can drain off the side of hills, preventing changes to hydrology, erosion and widening, whereas lower values mean trails more closely follow the fall line, which has the opposite effect (Marion and Wimpey 2017).

### Data Analysis

To determine if there were differences between formal and informal trails, paired t-tests were carried out on the following variables: elevation, Trail Grade, TSA, trail slope, width, maximum depth, soil loss, distance to nearest strata layers (ground cover, shrub and tree), soil compaction, canopy cover, erosion, root exposure, vegetation die-off, altered hydrology, presence of invasive species, presence of TTFs and overall trail condition.

Generalised Linear Models (GLMs) were conducted to identify variables that best predict trail width, trail depth, Cross-Sectional Area (CSA) of soil loss and overall trail condition (all, formal and informal trails). Variables were standardised before running GLMs. When trail width and trail depth were used as the dependent variables, Trail Grade, landform slope, TSA, difficulty and popularity were used as independent variables. For the GLM on soil loss, Trail Grade, trail slope, TSA, difficulty, popularity, width and TSR for the trail were used as dependent variables. Trail condition was used as the dependent variable and Trail Grade, trail slope, TSA, difficulty, popularity, width, depth, distance to ground cover and TSR were included as independent variables. As trail condition was correlated with all qualitative variables, they were excluded from the GLMs. All statistical analyses were carried out in R Studio (version 2023.09.1 + 494) (R Core Team 2023).

To determine if there were differences in soil loss between Trail Sustainability Ratings, Kruskal-Wallis non-parametric tests were carried out and Conover Iman post hoc tests were done after any significant results. A Spearman Rank Correlation test was performed on Trail Sustainability Ratings and overall trail condition to determine if there was a correlation between them.

To identify potential differences between trails of different difficulty ratings (Green, Blue and Black Diamond), a one-way ANOVA and Tukey’s post-hoc test and multiple Kruskal-Wallis tests and Conover Iman post-hoc tests when assumptions were violated were also conducted to compare soil loss, erosion, trail depth, trail width, Trail Grade and Trail Sustainability Rating in each difficulty category.

## Results

There was a total 31.4 km of formal (26 trails) and 33.7 km (28 trails) informal active mountain biking trails in Nerang National Park in 2023. This included 8.85 km (10 formal trails, 2 informal trails) of Green trails, 45 km (12 formal, 15 informal) of Blue trails, 9.65 km (4 formal, 8 informal) of Black Diamond trails while one 1 informal trail (1.5 km) did not have a difficulty rating on Trailforks. There were many TTFs recorded in the Park, with 45 TTFs recorded at or near the 80 points assessed.

### Environmental Impacts of Trails

Mountain bike trails in the Park had a range of environmental impacts including soil compaction and erosion, and the loss of vegetation on and adjacent to trails (Table 2). The trails averaged 0.9 m in width, and 5.5 cm in depth, with an average cross-sectional soil loss of 253 cm^2^ per point. There were edge effects from the trails including reductions in ground cover, shrub and tree cover and a slight reduction in canopy cover over the trails compared to 3 m off trail (Table 2).

**Table 2:** Mean and standard deviation along with maximum, minimum and *p* values for comparisons of factors between formal and informal mountain biking trails in Nerang National Park

Variable	Formal	Informal	*p* value*
Width (m) (min, max)	1.1 ± 0.7 (0.5–4.5)	0.7 ± 0.3 (0.4–1.5)	**0.001**
Depth (cm) (min, max)	4.6 ± 2.9 (1, 12)	6.3 ± 4.7 (0.5–25)	**0.040**
CSA soil loss (cm^2^) (min, max)	246 ± 186 (30, 850)	260 ± 301 (10, 1500)	0.820
Soil compaction (kg/cm^2^)	5.9	6	0.300
Trail Grade (%) (min, max)	7.9 ± 3.1 (2.8, 15.2)	8.2 ± 3.7 (5.2, 21.4)	0.700
Trail slope at point (%) (min, max)	6.2 ± 4 (0.5, 19)	8.2 ± 6.8 (1, 26.5)	0.120
TSA (^O^) (min, max)	60.1 ± 18.3 (25, 89)	60.9 ± 20.1 (10, 89)	0.840
Canopy cover loss (%)	4.3 ± 7.6	4.6 ± 7.1	0.980
Elevation (m) (min, max)	72.9 (19, 142)	127 (43, 230)	**<0.001**
Distance to ground cover (cm)	62.8	35.4	**0.001**
Distance to shrub (cm)	151.1	125.7	0.250
Distance to tree (cm)	206.6	195.9	0.650
Erosion (min, max)	6.7 (3, 10)	6.4 (1, 9)	0.600
Root exposure (min, max)	7.1 (3, 10)	6.7 (2, 10)	0.330
Vegetation die-off (min, max)	8.7 (6, 10)	8.5 (5, 10)	0.410
Altered hydrology (min, max)	7.9 (3, 10)	8.3 (5, 10)	0.390
Weeds present (min, max)	8 (4, 10)	7.9 (1, 10)	0.600
Trail condition (min, max)	8.1 (6.3, 8.8)	7.9 (5.2, 9.7)	0.420
Points with >1 TTF present	20	9	**0.006**

*CSA* cross-sectional area, *TSA* trail slope alignment, *TTF* trail technical feature

*P values from *t* tests. Bold indicates significant values.

Reflecting the steepness of much of the Park, trails tended to be sloped, and although the average slope at single points on the trails and average Trail Grade were within the sustainable levels (below 10%), some individual points and trails were much steeper (Table 2). Likewise, although the average alignment of trails (TSA) was within a desirable range (60°), at some points the values were much lower. The mainly natural, unhardened soil surface of the trails was generally highly compacted with an average of 6 kg/cm^2^ (Table 2).

Based on the width and depth of the trails it is possible to estimate the total amount of soil loss due to the trails for the whole Park. The average trail widths and depths for formal and informal trails were used to calculate this metric, with an estimate of 16.48 m^3^ of soil lost across the 65 km of mountain biking trails using the random point data. There was almost no difference in the estimated soil loss between formal and informal trails, which accounted for 47 and 53% of soil loss respectively (Table 3). Trail width and distance to ground cover were combined to calculate vegetation loss, which totalled 90,955 m^2^ across the Park with formal trails responsible for 61% and informal trails responsible for 39% (Table 3). There was also slightly less canopy cover on trails than off trails for the entire trail network (51% on, 55% off).

**Table 3:** Approximate soil loss, vegetation loss and canopy cover reduction across the mountain biking trail network in Nerang National Park

	Total trail network	Formal trails	Informal trails	Wetland catchment
Soil loss	16.48 m^3^	7.74 m^3^	8.76 m^3^	0.71 m^3^
Vegetation loss	90,955 m^2^	55,248 m^2^	35,707 m^2^	2898 m^2^

### Differences between Formal and Informal Trails

There were some differences between the two types of trails. Informal trails were concentrated in the higher elevation parts of the Park compared to formal trails (128 m vs 73 m) and further away from the lower altitude main entrance to the Park (Table 2, Fig. 1). In part because of this distance, informal trails were over six times less popular for mountain bikers than formal trails, with an average of 56 rides in six months for informal trail points compared to 372 for formal trail points. While none of the formal trails were in the RAMSAR wetland catchment area, 2.73 km of informal trails traversed the catchment (Fig. 1).

Other differences included formal trails being on average 1.6 times wider than informal trails, while informal trails were 1.4 times deeper (Table 2). This resulted in similar amounts of soil loss on average per point (253 cm^2^ per point). The other main difference between the two types of trails was the width to the ground cover layer which was 1.7 times greater on formal trails compared to informal trails (Table 2). There were more TTFs on formal trails than informal, in both presence at points (20 formal, 9 informal) and the number of individual features (33 formal, 12 informal).

### Trail Condition

The trail conditions measures were similar between formal and informal trails, indicating that some parts of the trail network in the Park were in poor condition. This included high erosion with exposed roots, evidence of vegetation die-off, altered hydrology and presence of weeds (Table 2). Erosion was the worst condition variable, with 24% of all points highly eroded (scores 1–4) and an average value of 6.5 (Table 2). Roots were often exposed on trails, with 17% of all points rated poor for root exposure and an average of 6.9 across the entire trail network (Table 2). There were more invasive weeds present on informal trails (17% poor) than formal trails (3% poor) but overall, the trails had an average rating of 8.3 (Table 2). Trail points scored better overall for both altered hydrology (average 7.9) and vegetation die-off (average 8.6) (Table 2). This led to the overall trail network having a ‘good’ average rating of 8, with the lowest overall trail condition rated 5.2 and the highest 9.7 (Table 2).

### Factors Affecting Trail Condition

Results from GLMs show that popularity and alignment (TSA) best-predicted trail width, where both more popular trails and worse alignment (lower TSA) resulted in wider trails (Table 4). The slope and alignment of trails best-predicted trail depth, with higher Trail Grades and steeper slopes resulting in deeper trails while worse aligned trails (lower TSA) resulted in deeper trails. Slope (increase), alignment (TSA, decrease) and width (increase) of trails best-predicted soil loss (CSA) on trails (Table 4). For trail condition, the important predictors were trail depth (deeper trails = worse condition), popularity (more popular = worse condition), trail slope (steeper slopes = worse condition) and alignment (worse/lower TSA = worse condition) across the mountain biking trails in the Park. There were slight differences in the best predictors for trail condition between formal and informal trails (Table 4), with popularity, trail slope and TSA best predictors for formal trails, while trail depth, trail slope and TSA best predictors for informal trails (Table 4).

**Table 4:** Results from Generalised Linear Models showing models that best predict width, depth and soil loss of all trails and trail conditions (average of qualitative variables recorded at each trail point) for all trails, then formal and informal trail separately with odds ratios, *p* values and *R*^2^ for each model

Independent variable	Explanatory variables (odds ratio)	*p* values	*R*^2^
Trail width – all trails	**Popularity (1.4)**, TSA (0.8)	**0.005**, 0.1	0.20
Trail depth – all trails	Trail Grade (1.2), **trail slope (1.7), TSA (0.8)**	0.12, **<0.001, 0.01**	0.47
Soil loss – all trails	**Trail Grade (1.6), TSA (0.8), width (1.2)**	**<0.001, 0.004, 0.05**	0.43
Trail condition – all trails	**Depth (0.8), popularity (0.9), trail slope (0.7), TSA (1.4)**	**0.03,** **0.07,** **<0.001,** **<0.001**	0.56
Trail condition – formal	**Popularity (0.8), trail slope (0.7), TSA (1.3)**	**0.02,** **<0.001,** **0.001**	0.51
Trail condition – informal	**Depth (0.7), trail slope (0.7)**, TSA (1.3)	**0.04,** **0.02**, 0.07	0.59

*P* values in bold indicate significant values

*TSA* trail slope alignment

### Sustainability Rankings of Trails

The sustainability of trails was assessed as Good, Neutral, Poor and Very Poor using Trail Sustainability Ratings based on Trail Grade and Alignment (TSA). Much of the formal and informal trail networks (75% of trails) were rated as Good using this matrix (Table 5). However, there were trails that were in poor condition, with 20% of formal and 17.5% of informal trails rated poor and 2.5% of formal and 7.5% of informal trails rated very poor (Table 5). Trail Sustainability Ratings based on grade and alignment were closely related to the condition of trails showing that trails with bad alignment and grades associated with decreased trail condition (*p* < 0.001, *R*^2^ = 0.16). On the formal trails, there was no correlation between the sustainability of trails based on Trail Sustainability Rating and the amount of soil loss (*p* = 0.210) in contrast to informal trails, where there was soil loss was higher for trails with poorer ratings (*p* = 0.003, Table 5).

**Table 5:** Number of formal and informal mountain biking trail points surveyed by Trail Grade and Trail Slope Alignment, percentage of trails per Trail Sustainability Ratings, and results of Kruskal-Wallis tests comparing formal and informal mountain biking trails in Nerang National Park

Study Area	Trail Slope Alignment	Trail Grade				Totals
		0–2%	3–10%	11–20%	>20%	
Formal Informal	0–30			1		1
			4	1	2	7
Formal Informal	31–60		15	6		21
			10	1		11
Formal Informal	61–90	1	15	2		18
			20	2		22
Formal Informal	Totals	1	30	9	0	40
		0	34	4	2	40

Study Area		Trail Sustainability Ratings				
		Good	Neutral	Poor	Very Poor	
Formal		75.0%	2.5%	20.0%	2.5%	
Informal		75.0%	0.0%	17.5%	7.5%	
Chi-squared, df		Mean CSA Soil Loss (cm^2^)				Sig
4.53, 3	Formal	239	76	294	426	0.210
11.77, 2	Informal	170	–	274	1131	**0.003**

Bold indicates significant values

The difficulty rating of trails was associated with environmental impacts and poorer trail conditions (Table 6). Black Diamond trails had more erosion and were steeper (in terms of both Trail Grade and trail slope at individual points) than both Blue and Green trails, were deeper and had more soil loss than Green trails (Table 6). However, somewhat surprisingly, trails did not significantly vary in width or TSA between difficulty ratings (Table 6).

**Table 6:** *P* values from Kruskal-Wallis tests and Conover Iman post-hoc tests comparing soil loss, erosion, trail depth, trail width, Trail Grade, Trail Slope Alignment (TSA) and Trail Sustainability Rating (TSR) between mountain biking trail difficulty ratings

	Overall	Green vs Blue	Green vs Black Diamond	Blue vs Black Diamond
Soil loss*	**0.028**	0.529	**0.046**	0.058
Erosion	**0.018**	0.194	**0.017**	**0.015**
Depth	**0.035**	0.055	**0.014**	0.068
Width	0.093	–	–	–
Trail slope	**<0.001**	**0.006**	**<0.001**	**0.0007**
Trail Grade	**<0.001**	**0.0001**	**<0.001**	**0.0007**
TSA	0.34			
TSR	**0.002**	0.437	**0.015**	**0.0006**

*Soil loss *p* values from one-way ANOVA and Tukey’s post hoc tests using logged values. Bold indicates significant values

## Discussion

Extensive networks of mountain biking trails, such as those in Nerang National Park, have a range of negative environmental impacts. The amount of use trails receives and different design features, such as slope and alignment, affect the severity of impacts. These results have important implications in terms of understanding the environmental impacts of mountain biking trails and how to minimise them. This is especially relevant in the context of increasing demand for mountain biking and the still sparse research assessing environmental impacts of this activity (Pickering 2022), including studies directly comparing formal and informal mountain bike trails (Ballantyne and Pickering 2015a).

### Environmental Impacts of Formal and Informal Trails

There were some important differences between the two types of trails and their impacts in Nerang National Park. On average, formal trails were wider than informal trails, while informal trails were deeper. Differences in trail widths are associated with many factors, including design, activity type, difficulty and use (Webber et al. 2004). For example, formal trails may be wider for safety reasons, for easier movement or to allow easier passing by different users on trails (Webber et al. 2004; Wimpey and Marion 2011; Carsten 2023). Informal trails on the other hand are not designed with sustainable trail building in mind and may receive less use (Wimpey and Marion 2011). Overall, in Nerang National Park, which is relatively steep in parts, the widths of the formal and informal trails (1.3 m vs 0.71 m) were narrower compared to trails through flatter areas of urban remnant forest in the same region (2.9 m formal vs 1.5 m informal) (Ballantyne and Pickering 2015b). Furthermore, increased trail widths result in greater vegetation loss from formal than informal trails in Nerang National Park, as found in other urban remnant forests in South-East Queensland (Ballantyne and Pickering 2015b). However, as informal trails extend the total extent of trails and often traverse sensitive ecosystems, they can be more damaging, contributing to additional habitat loss, degradation and fragmentation (Leung et al. 2011; Runkowski 2016).

In Nerang National Park, mountain biking trails were associated with soil loss, which is common with poorly aligned unhardened trails, particularly on steep slopes (Ballantyne and Pickering 2015b; Runkowski 2016; Marion and Wimpey 2017; Meadema et al. 2020). With formal trails wider but shallower than informal trails, they had similar amounts of cross-sectional soil loss overall (formal 246 cm^2^, informal trails 260 cm^2^), although there was considerable variation with extensive erosion observed on some trail sections. Trail design affects soil loss, with poor and very poorly designed trails experiencing far more soil loss (Good TSR = 205 cm^2^, Very Poor TSR = 779 cm^2^). Marion and Wimpey (2017) also found that poorly designed trails with low Trail Sustainability Ratings experienced greater levels of soil loss on formal mixed-use trails in the United States, although the average amount of cross-sectional soil loss there was a lot higher compared to the Park (Good TSR = 636 cm^2^, Very Poor TSR = 1370 cm^2^).

### Trail Condition and Sustainability of Trails

Approximately 60% of both formal and informal trails were in good condition in Nerang with an overall trail condition rating of eight or above. Furthermore, 75% of both formal and informal trails had a rating of Good in the Trail Sustainability matrix, which is comparative to results observed in Marion and Wimpey (2017) where trail networks had between 48% and 83% of trails rated Good. However, the condition of the remaining sections of trails in Nerang National Park is of concern, with some low values for individual measures of trail condition, such as erosion, altered hydrology, root exposure and presence of weeds.

The technical difficulty of trails was associated with differences in trail conditions in Nerang, something not often addressed in the literature (Hemanth et al. 2021). In the Park, Black Diamond trails had worse trail conditions and were both deeper and steeper, and more environmental impacts, including greater erosion and more soil loss than other trail types. This is most likely due to Black Diamond trails needing to be technically challenging and often including sections of trail that are downhill and very steep (Carsten 2023). Less of a difference in design standards may explain Blue and Green trails being more similar in condition and environmental impacts, with trail slope and Trail Grade the only differences (Carsten 2023).

### What Predicts Environmental Impacts and Trail Condition?

The design and location of trails, including slope, soil type, climate, landform and alignment, influence the overall condition of trails and their environmental impacts (Spernbauer et al. 2023). They can affect trail width, depth, soil loss and erosion, and vegetation loss, as seen in the Park, as well as other areas in the region (Ballantyne and Pickering 2015b; Runkowski 2016) and internationally (Tomczyk 2011; Marion and Wimpey 2017; Meadema et al. 2020; Spernbauer et al. 2023; Fang and Ng 2024). Steeper slopes, both overall and at individual points along trails, were associated with narrower but deeper trails, more soil loss and worse trail condition scores. Conversely, when the trail alignment angle was higher (trails perpendicular to the fall line), trails were shallower with less soil loss, and the overall condition of trails were good. Trail slope and Trail Grade had the greatest influence on the environmental impacts and condition of mountain biking trails compared to other variables analysed, both in this and other studies (Meadema et al. 2020; Spernbauer et al. 2023; Fang and Ng 2024).

Trail use has previously been shown to influence trail widening and other trail impacts, with the widest trails experiencing the most traffic and often close to entrances and specific attractions (Tomczyk et al. 2017; Meadema et al. 2020). This was also the case in this study, where trails that were more popular were both wider and had poorer overall trail condition, particularly on the formal network. There was a lot more variation in the amount of use between formal trails compared to informal trails, as well as formal trails being six times more popular overall than informal trails, leading to trail popularity influencing the condition and environmental impact of formal trails.

### Management Recommendations and Further Research Opportunities

Nerang National Park has a substantial issue with an informal trail network accounting for over 50% of trails. These informal trails extend the overall trail network, subsequently increasing soil and vegetation loss (Leung et al. 2011; Ballantyne et al. 2014). The results also highlight the importance of good design in the creation of trails, with trails with poor alignment on steep slopes in poorer condition.

There are some formal trails in poor condition due to sections with steep slopes and poor trail alignment. Therefore, a review of these trails is required to examine the feasibility and desirability of realignment. Realignment of trails can include altering them to be side-hill trails, following contours instead of fall lines, or including more switches in alignment to limit water flow along the trails and reduce environmental impacts (Meadema et al. 2020). Realignment of trails can be both challenging and costly, however, can save resources in the long term by reducing maintenance requirements as well as reducing environmental impacts such as soil erosion (Olive and Marion 2009; Meadema et al. 2020). Designing and constructing strategically placed pass points, reinforcing trail edges with rocks or logs and active management are ways to further minimise impacts on formal trails with high use (Runkowski 2016).

As some of the informal trails are well designed, and in good condition with lower environmental impacts, there may be the option to convert some of the most sustainable informal trails into formal trails (Pickering et al. 2010). Converting informal trails into formal trails could increasing the trail network in the Park overall, or they could replace current formal trails in poorest condition, depending on the goals of the land managers. The conversion of informal trails into formal trails can assist in addressing unmet demand for mountain biking trails and enhance relationships between land managers and local mountain biking groups and riders if these stakeholders are included in the process (Pickering et al. 2010).

It is recommended that most of the informal trails be reviewed with consideration to their closure and rehabilitation, however, starting with the trails traversing the RAMSAR-listed wetland catchment area and those with the poorest overall trail condition rating (Spernbauer et al. 2023). Closing informal trails and rehabilitating the forest will reduce vegetation loss, soil loss and other environmental impacts. Other research has made similar recommendations regarding informal trails damaging the environment and located in sensitive areas (Ballantyne et al. 2014; Lucas 2020). Environmental benefits from closing trails, however, will only accrue if the trails remain closed and new ones are not created (Hockett et al. 2017). This study has shown how land managers of a national park can utilise publicly available volunteered geographic information data to monitor the use of informal and closed trails. This can enable land managers to understand if their efforts to close and rehabilitate trails are being successful or not, something that has also been demonstrated previously across an entire region (Loosen et al. 2023).

The construction and use of informal trails is an issue in South-East Queensland (Ballantyne et al. 2014; Ballantyne and Pickering 2015b) but also elsewhere around the world (Leung et al. 2011; Barros et al. 2013; Hardiman and Burgin 2013). Informal trails are often built and used because visitors’ needs are not being met in some way (Newsome and Davies 2009; Marion 2023). For mountain bikers, this is often that trails are not challenging or diverse enough, too busy or riders want a shortcut (Newsome and Davies 2009; Leung et al. 2011; Ballantyne and Pickering 2015b; Havlick et al. 2016). Preventative methods to reduce informal trail use and creation include encouraging riders to use formal trails either in the Park or other areas, physically blocking informal and closed trails and informative signage regarding not using informal and closed trails (Ballantyne and Pickering 2015a; Hockett et al. 2017). Collaborating with relevant stakeholders on what trail features mountain bikers want and their motivations can also be beneficial to land managers. This knowledge can assist in designing other trails, moving people away from high conservation value areas and preventing new informal trails being created (Ballantyne and Pickering 2015a; Newsome et al. 2016). Well-designed trails or mountain biking-specific parks catered to many different groups can then be built in areas not already designated for conservation, such as private property, old farmland or State Forests (Hardiman and Burgin 2013; Ballantyne and Pickering 2015a).

## Conclusion

This study highlights some important differences between formal and informal mountain biking trails, including that formal trails were wider and more popular, and informal trails were deeper and mostly in more remote areas. All trails had impacts, including soil loss, erosion and vegetation loss, however, well-designed trails were in better condition, were more sustainable and minimised environmental impacts. The informal trail network in Nerang National Park is extensive, highlighting a lack of mountain biking infrastructure in the region, something that has been observed elsewhere, posing a challenge for managing the Park at a local scale. More broadly, these findings demonstrate how more mountain biking facilities are needed in areas not already designated for conservation, and trails need to be designed correctly to minimise environmental impacts and ensure their continuing sustainability.

## Data Availability

Data are provided within the manuscript or supplementary information files.
